# Long‐Term Immunogenicity, Safety, and Reactogenicity of CoronaVac and Heterologous Booster Vaccines: A Two‐Year Cohort Study in Brazil

**DOI:** 10.1155/cjid/3683298

**Published:** 2026-11-09

**Authors:** Sarah Vieira Contin, B. F. Nathalie Almeida, S. Priscilla Filgueiras, A. Camila Corsini, A. P. Daniel de Miranda, J. Wander de Jeremias, A. Olindo Martins-Filho, Andrea Teixeira-Carvalho, Ana Carolina Campi-Azevedo, A. Dayane Otta, G. Jaquelline de Oliveira, R. F. Guilherme Campos, L. Mauricio Nogueira, Pedro Augusto Alves, S. Thaís Bárbara de Silva, R. Gabriel Fernandes, R. Leda Castilho, S. Priscila Fernanda Martins, J. Adelina Lourenço, V. Jessica de Assis, L. Érica Oliveira, S. Ana Esther de Lima, M. Tulio Lima, P. B. Daniel de Abreu, G. F. Renata Alvim, F. Q. Rafaella Grenfell

**Affiliations:** ^1^ Diagnosis and Therapy of Infectious Diseases and Cancer Oswaldo Cruz Foundation (FIOCRUZ) 1715 Augusto de Lima Avenue, Minas Gerais Belo Horizonte 30190 002 Brazil fiocruz.br; ^2^ Experimental Pharmacology Laboratory College of Pharmacy Federal University of Ouro Preto (UFOP) 306-328 Três Street, Minas Gerais Ouro Preto 35400-000 Brazil ufop.br; ^3^ Integrated Biomarker Research Group Oswaldo Cruz Foundation (FIOCRUZ) 1715 Augusto de Lima Avenue, Minas Gerais Belo Horizonte 30190 002 Brazil fiocruz.br; ^4^ Cellular and Molecular Immunology Laboratory Oswaldo Cruz Foundation (FIOCRUZ) 1715 Augusto de Lima Avenue, Minas Gerais Belo Horizonte 30190 002 Brazil fiocruz.br; ^5^ Virology Research Laboratory (LPV) São José do Rio Preto Medical School (FAMERP) 5416 Brg. Faria Lima Avenue, Vila São Pedro, São Paulo São José do Rio Preto 15090-000 Brazil; ^6^ Base Hospital 5544 Brg. Faria Lima Avenue, Vila São José, São Paulo São José do Rio Preto 15090-000 Brazil; ^7^ Department of Pathology University of Texas Medical Branch 301 University Boulevard, Texas Galveston 77555-0144 USA utmb.edu; ^8^ Immunology of Viral Diseases Oswaldo Cruz Foundation (FIOCRUZ) 1715 Augusto de Lima Avenue, Minas Gerais Belo Horizonte 30190 002 Brazil fiocruz.br; ^9^ Cell Culture Engineering Laboratory (COPPE) Universidade Federal do Rio de Janeiro 2030 Horácio Macedo Avenue, Cidade Universitária da Universidade Federal do Rio de Janeiro Rio de Janeiro 21941-598 Brazil ufrj.br; ^10^ Department of Infectious Diseases College of Veterinary Medicine University of Georgia 501 DW Brooks Drive, Georgia Athens 30602 7387 USA uga.edu

**Keywords:** antibodies, cytokines, vaccination, viral

## Abstract

This two‐year cohort study evaluated immunogenicity, safety, and reactogenicity in 1675 Brazilian healthcare workers who received CoronaVac as a primary protocol (PP) followed by heterologous boosters. All vaccines were safe, with no serious adverse events. Reactogenicity was highest for Ad26.COV2.S (62.86%) as a second booster, followed by Covishield (58.56%) and Comirnaty (42.62%); CoronaVac PP reactogenicity was 19.58%. Total anti‐S IgG levels remained high throughout, increasing after the first booster. At 9 months post‐PP, Comirnaty‐boosted individuals showed superior responses versus CoronaVac alone, regardless of prior infection. No differences were observed between one or two boosters, age groups, or sex. Initially, previously infected participants had higher anti‐S IgG (6–9 months), but this difference disappeared post‐booster. Neutralizing antibodies against Omicron BA.1 were stronger in previously infected individuals at 6–12 months. The first booster increased neutralization in both groups; the second booster further enhanced responses in infection‐naive individuals and maintained them in previously infected participants, with sustained activity up to 24 months. Participants with prior COVID‐19 showed higher levels of immunological biomarkers early on, but this gap closed after the second booster, which elevated biomarkers in both groups. Cellular response, inferred from biomarker interactions, was generally higher in infection‐naive individuals. These results underscore the importance of heterologous booster regimens in sustaining long‐term humoral and cellular immunity against COVID‐19.

## 1. Introduction

Vaccines play a crucial role in disease prevention and control, as well as in monitoring the immune response they elicit. [1]. However, the rapid development of COVID‐19 vaccines and the lack of long‐term postvaccination follow‐up highlight the need for continuous monitoring to determine the extent to which the immunological response elicited by vaccination diminishes or persists, as well as the optimal timing for administering booster doses [2].

On January 17, 2021, the Brazilian National Health Surveillance Agency (Anvisa) authorized the emergency use of CoronaVac (Sinovac/Instituto Butantan) in Brazil [3]. By May 2021, vaccination progress had yielded promising outcomes, with a study from the Federal University of Pelotas (UFPel‐RS) reporting a 50% reduction in mortality among individuals aged 80 and older [4].

Following the approval of CoronaVac, other vaccines were approved for use in Brazil. Comirnaty was granted full regulatory approval on February 23, 2021 [5], followed by Covishield on March 12, 2021 [6], and Ad26.COV2.S on April 5, 2022 [7]. In addition, the first and second COVID‐19 booster doses were approved in Brazil in October 2021 and June 2022, respectively, further expanding the national vaccination strategy [8, 9].

In December 2021, a study commissioned by the Brazilian Ministry of Health (MS) demonstrated that heterologous booster regimens were more effective than homologous ones in individuals who had received the CoronaVac primary protocol (PP), with all heterologous combinations eliciting superior anti‐S IgG responses [10]. Based on these findings, the MS recommended the Comirnaty (Pfizer‐BioNTech) vaccine for booster doses. This decision marked a key milestone in Brazil’s vaccination strategy, supported by evidence showing that vaccine‐induced humoral responses wane over time, while booster doses significantly enhance immunogenicity [11].

A study demonstrated that individuals vaccinated against SARS‐CoV‐2 retained cellular immunity for up to 20 months following their last exposure to the SARS‐CoV‐2 Spike antigen [12]. Despite this, the long‐term cellular immune response remains insufficiently understood. Additionally, the emergence of more infectious SARS‐CoV‐2 variants that may reduce vaccine efficacy reinforces the need for ongoing research to confirm the long‐term safety and effectiveness of existing vaccines [1].

The present study, therefore, evaluated the humoral and cellular responses, as well as the safety of the vaccination protocol over a two‐year period. This included identifying positive cases of COVID‐19 postvaccination among healthcare professionals at two hospitals who received two doses of the inactivated virus vaccine CoronaVac, followed by heterologous booster doses.

## 2. Materials and Methods

### 2.1. Study Design and Ethics

This cross‐sectional study was reviewed and approved by the National Research Ethics Commission (CONEP: CAAE 30428720.3.0000.5091) and was conducted from March 2021 to March 2023. A total of 1.675 professionals from Hospital da Baleia and Hospital Metropolitano Doutor Célio de Castro, both in Belo Horizonte, Minas Gerais, Brazil, participated after signing the informed consent form (ICF). All participants completed the primary vaccination protocol with two doses of CoronaVac, followed by booster doses of Ad26.COV2.S (Janssen/Johnson & Johnson), Covishield (Oxford/Astrazeneca/Fiocruz), or Comirnaty (Pfizer‐BioNTech). In accordance with authorizations and guidelines issued by the MS, the vaccination protocols administered to participants followed the national immunization plan, which included healthcare professionals among the priority groups.

After consent, participants completed epidemiological questionnaires, addressing demographics, comorbidities, vaccination history, adverse events, and SARS‐CoV‐2 infection status before or after CoronaVac PP. Participants were closely monitored throughout the study by the clinical team. Those reporting flu‐like symptoms (cough, fever, headache, myalgia) underwent nasopharyngeal swab collection for SARS‐CoV‐2 testing by reverse transcription real‐time polymerase chain reaction (RT‐qPCR). Positive samples were further analyzed via next‐generation sequencing (NGS) [1].

### 2.2. Inclusion Criteria

Healthcare workers from Hospital da Baleia and Hospital Metropolitano Doutor Célio de Castro, Belo Horizonte, Minas Gerais, Brazil, who had completed the primary vaccination regimen consisting of two doses of CoronaVac, regardless of subsequent booster dose administration, and who agreed to participate in the study and signed the ICF were eligible for inclusion.

### 2.3. Sample Collection

Approximately 5 mL of peripheral blood was collected from participants by venipuncture at 6, 9, 12, 15, 18, 21, and 24 months after the second CoronaVac dose. Blood collection was performed on predefined dates for each follow‐up visit: 6 months (July 28–29 and August 11–12, 2021), 9 months (November 18–19, 2021), 12 months (February 10–11 and 17–18, 2022), 15months (May 5–6 and 12–13, 2022), 18 months (August 3–4 and 11–12, 2022), 21 months (November 17–18 and 24–25, 2022), and 24 months (February 23–24 and March 2–3, 2023).

All blood samples underwent analysis for anti‐S IgG ELISA, Luminex Bio‐Plex Pro human cytokine platform to determine the immunological biomarker profile, and viral microneutralization assay (VNT) (Supporting figure 1).

### 2.4. Extraction and Purification of SARS‐CoV‐2 RNA

The QIAamp Viral RNA Kits were used for extraction and purification of SARS‐CoV‐2 RNA according to the manufacturer’s instructions. Briefly, 140 μL of viral transport medium (VTM) from nasopharyngeal swabs was mixed with 560 μL of AVL buffer containing carrier RNA, vortexed, incubated for 10 min at RT, and combined with 560 μL of ethanol. The mixture was applied twice (630 μL each) to a QIAamp Mini column, centrifuged at 6000 × g for 1 min, and washed sequentially with 500 μL AW1 and 500 μL AW2 buffers (20,000 × g, 3 min). Viral RNA was eluted with 60 μL AVE buffer after 1 min incubation at RT and stored at −80°C.

### 2.5. RT‐qPCR

The purified RNA was subjected to RT‐qPCR using the Charité/Berlin protocol, which detects amplification of the SARS‐CoV‐2 *E* gene, and the Centers for Disease Control and Prevention (CDC, EUA) protocol, which detects amplification of two regions of the SARS‐CoV‐2 N gene (N1 and N2) [13]. The RT‐qPCR reaction was performed with the GoTaq Probe 1‐Step RT‐qPCR System kit (Promega).

For the Charité/Berlin protocol, the reaction mix included 10 μL of GoTaq Probe qPCR Master Mix with dUTP 2*X* (Promega), 0.4 μL of GoScript RT Mix 1‐step RT‐qPCR (Promega), 2 μL of 2019‐nCoV gene *E* [Integrated DNA Technologies (IDT)], 0.4 μL of 10 mM RNAse P forward and reverse primer (IDT), 0.3 μL of 10 mM RNAse P probe (IDT), and 1.5 μL of nuclease‐free water. For the CDC protocol, the reaction mix included 10 μL of GoTaq Probe qPCR Master Mix with dUTP 2*X* (Promega), 0.4 μL of GoScript RT Mix 1‐step RT‐qPCR (Promega), 1.5 μL of primer/probe mix for N1, N2, or RP (Kit 2019‐nCoV RUO – IDT), and 3.1 μL of nuclease‐free water. Thermocycling conditions were as follows: reverse transcription at 45°C for 15 min, followed by initial denaturation at 95°C for 2 min, and 40 cycles of 95°C for 15 s and 60°C for 1 min. The QuantStudio 12 K Flex Real‐Time PCR system or the ViiA7 Real‐Time PCR system was used to perform the assays.

### 2.6. Detection of anti‐SARS‐CoV‐2 IgG Antibodies (ELISA)

The S protein of the SARS‐CoV‐2 virus was expressed in stable recombinant HEK293 cells, as described in the study by Alvim et al. [14]. It was produced by the Federal University of Rio de Janeiro (UFRJ) and used as the antigen in the ELISA assay for the detection of total IgG antibodies against SARS‐CoV‐2. For this assay, 96‐well plates (Corning) were coated with 200 ng/well of purified spike protein in 50 μL/well of phosphate‐buffered saline (PBS), composed of NaCl, KCl, Na_2_HPO_4_, and KH_2_PO_4_, pH 7, overnight at 4°C. Next, the plates were washed five times with PBS and blocked with 2% milk protein diluted in PBS for 1 h and 30 min at room temperature (RT). The plates were washed again, and serum samples diluted 1:40 in 1% blocking buffer 50 μL per well—were added and incubated for 2 h at RT. The plates were washed again, followed by a 90 min incubation with a 1:60,000 dilution in PBS of the HRP‐conjugated anti‐human IgG secondary antibody (Sigma‐Aldrich). After another wash, the reaction was initiated by adding 50 μL/well of 3,3′,5,5′‐tetramethylbenzidine (TMB) (Sigma‐Aldrich) for 10 min and stopped by adding 25 μL/well of 1N H_2_SO_4_. The optical density (OD) was read at 450 nm using the Multiskan FC microplate reader (Thermo Fisher).

### 2.7. Determination of Immunological Biomarker Profile

The cellular immune response was assessed by quantifying soluble mediators in serum using the Luminex Bio‐Plex Pro human cytokine platform, in collaboration with the Fiocruz Minas Flow Cytometry Platform. Serum samples (23–37 per time point and subgroup) were collected at 6, 9, 12, 15, 18, 21, and 24 months post‐CoronaVac PP from individuals with and without prior SARS‐CoV‐2 infection. The assay evaluated: chemokines (CXCL8, CCL11, CCL3, CCL4, CCL2, CCL5, and CXCL10); proinflammatory cytokines (IL‐1b, IL‐6, TNF‐a, IL‐12, IFN‐g, IL‐15, and IL‐17); regulatory cytokines (IL‐1ra, IL‐4, IL‐5, IL‐9, IL‐10, and IL‐13); and growth factors (FGF‐basic, PDGF, VEGF, G‐CSF, GM‐CSF, IL‐7, and IL‐2). Serum samples were thawed in a water bath at 37°C, homogenized by vortexing for 5 s, and centrifuged at 14.000 × g for 5 min at RT. Subsequently, 50 μL of the samples were added to previously prepared Luminex assay plates by adding 50 μL of the bead mixture (diluted 10 ×) to each well, followed by washing (twice with 100 μL of wash buffer). The plates were incubated overnight at 4°C under shaking (300 ± 50 rpm). The following day, 25 μL of detection antibody (previously diluted 10 ×) was added to each well and incubated for 30 min on a shaker (300 ± 50 rpm) at RT, protected from light. Then, washing was conducted with 100 μL of wash buffer (3 times). Subsequently, 50 μL of streptavidin‐phycoerythrin (SAPE) (previously diluted 10 ×) was added to each well and incubated for 30 min on a shaker (300 ± 50 rpm) at RT, protected from light. After that, washing was done with 100 μL of wash buffer. The pellet was resuspended with the addition of 125 μL of assay buffer, and the samples were homogenized for 10 min. The mean fluorescence intensities were determined by acquiring the samples (50 microspheres per analyzed analyte) on the Bio‐Plex 200 equipment (Bio‐Rad Laboratories, California, USA), using the Luminex xPONENT software version 3.1 (Merck Millipore).

### 2.8. VNT

The VNT was developed in partnership with the São José do Rio Preto School of Medicine (Famerp) to determine the levels of neutralizing antibodies against variants of concern at all time points ever described. The test was conducted in an NB3 laboratory at Famerp, and for each time point analyzed, 15 samples were selected from individuals with COVID‐19 prior to vaccination and another 15 samples from individuals without COVID‐19 infection. In this assay, on the day prior to the experiment, 104 Vero cells were seeded into 96‐well plates with transparent flat bottoms and incubated for 24 h at 37°C and 5% CO_2_, reaching 90%–95% confluence after incubation. The serum samples were heat‐inactivated for 30 min at 56°C and subsequently diluted in fresh DMEM media supplemented with 100 U/mL penicillin, 100 μg/mL streptomycin, and 2% fetal bovine serum. For each serum sample, eight dilutions were tested (1:20, 1:40, 1:80, 1:160, 1:320, 1:640, 1:1280, and 1:2480). The diluted samples were incubated with the live SARS‐CoV‐2 variant at a constant concentration of 50 TCID50/mL at 37°C for 1 h. Finally, the samples were seeded into Vero CCL‐81 cell plates and maintained in a humidified incubator at 37°C and 5% CO_2_ for 72 h. The cells were fixed with 10% formaldehyde for 20 min and stained with crystal violet. Neutralizing capacity was determined by the presence or absence of a cytopathic effect across the dilutions, and for each sample, the serum dilution corresponding to 50% virus neutralization (VNT50) was determined. All samples, at all dilutions, were tested in triplicate, and the VNT50 was calculated using the Spearman–Karber algorithm. To validate the assay, a serum sample known to neutralize the Omicron (BA.1) variant and a sample collected before the pandemic were used as positive and negative controls, respectively.

### 2.9. Data Analysis

Data analyses were performed using GraphPad Prism (v8.0) and Cytoscape. RT‐qPCR results were processed via the Thermo Cloud platform. Demographic variables were summarized using descriptive statistics. Age was categorized into predefined age groups for all analyses, and sex was treated as a categorical variable. Quantitative immunological data, including ELISA, VNT50, and Luminex‐derived parameters, were assessed for normality using the Kolmogorov–Smirnov test. As the data did not follow a normal distribution, nonparametric statistical tests were applied. Comparisons of antibody responses and Luminex‐derived parameters according to demographic and clinical variables (age group, sex, and previous SARS‐CoV‐2 infection status) were performed using the Mann–Whitney *U* test for two‐group comparisons and the Kruskal–Wallis test for comparisons involving more than two groups. Biomarker concentrations were calculated from standard curves generated with a 5‐parameter logistic regression model using Bio‐Plex Manager software (v6.1, Bio‐Rad). Serum levels of soluble mediators, originally expressed as continuous variables (pg/mL), were converted into categorical data (percentage proportion) using global median values as cut‐off points to identify the proportion of individuals with elevated mediator levels. Statistical significance was set at *p* < 0.05.

## 3. Results

### 3.1. Epidemiological Data

A total of 1.675 individuals were included. All of them were vaccinated with CoronaVac PP. Regarding the first booster, 52.18% (874/1675) participants received Comirnaty, 1.13% (19/1675) Covishield, and 0.60% (10/1675) Ad26.COV2.S. For the second booster, 7.42% (67/903) participants received Comirnaty, 20.93% (189/903) Covishield, and 4.21% (38/903) Ad26.COV2.S (Supporting figure 2).

Overall, 45.43% (761/1675) received only CoronaVac PP, 35.22% (590/1675) CoronaVac PP + Comirnaty as first booster, 3.64% (61/1675) CoronaVac PP + Comirnaty as first and second booster, 2.09% (35/1675) CoronaVac PP + Comirnaty as first booster + Ad26.COV2.S as second booster, and 10.80% (181/1675) CoronaVac PP + Comirnaty as first booster + Covishield as second booster. Demographic and clinical characteristics, as well as prior and postvaccination COVID‐19 infection, are summarized for each group in Table 1.

**TABLE 1:** Epidemiological stratification of participants.

	CoronaVac PP (*n* = 761)	CoronaVac PP + Comirnaty (*n* = 590)	CoronaVac PP + Comirnaty + Comirnaty (*n* = 61)	CoronaVac PP + Comirnaty + Ad26.COV2.S (*n* = 35)	CoronaVac PP + Comirnaty +Covishield (*n* = 181)
Age^1^ (mean + SD)	36.8 ± 10.0	38.2 ± 9.7	41.6 ± 10.5	42.7 ± 10.8	40.2 ± 9.9
Male, *n* (%)	192 (25.23)	140 (23.73)	7 (11.47)	7 (20.00)	37 (20.44)
SARS‐CoV‐2 infection prior to vaccination, *n* (%)	131 (17.21)	75 (12.71)	4 (6.56)	3 (8.57)	18 (9.94)
SARS‐CoV‐2 infection postvaccination, *n* (%)	105 (13.80)	138 (23.39)	26 (42.62)	12 (34.28)	73 (40.33)
Hypertension, *n* (%)	70 (9.20)	64 (10.85)	10 (16.39)	7 (20.0)	25 (13.81)
Obesity, *n* (%)	42 (5.52)	35 (5.93)	3 (4.92)	3 (8.57)	11 (6.08)
Smoking, *n* (%)	30 (3.94)	27 (4.58)	2 (3.28)	1 (2.86)	5 (2.76)
Asthma, *n* (%)	27 (3.55)	24 (4.07)	1 (1.64)	2 (5.71)	10 (5.52)
Hypothyroidism, *n* (%)	23 (3.02)	16 (2.71)	6 (9.84)	1 (2.86)	5 (2.76)
Diabetes, *n* (%)	15 (1.97)	14 (2.37)	2 (3.28)	3 (8.57)	7 (3.87)

*Note:* Absolute values and percentages of demographic and clinical characteristics of participants categorized by vaccination protocol and prior health conditions.

^1^mean + standard deviation.

### 3.2. Reactogenicity

The most reported adverse events following immunization (AEFI) were local pain, myalgia, headache, malaise, fever, and chills. The proportion of participants experiencing each AEFI varied across vaccination groups, with some participants reporting no events. Detailed AEFI data for each group are presented in Table 2.

**TABLE 2:** Adverse events following different COVID‐19 vaccination protocols.

	CoronaVac PP (*n* = 761)	CoronaVac PP + Comirnaty (*n* = 590)	CoronaVac PP + Comirnaty +Comirnaty (*n* = 61)	CoronaVac PP + Comirnaty + Ad26.COV2.S (*n* = 35)	CoronaVac PP + Comirnaty + Covishield (*n* = 181)
Absence of AEFI, *n* (%)	612 (80.42)	285 (48.30)	35 (57.38)	13 (37.14)	75 (41.44)
Local pain, *n* (%)	27 (3.55)	169 (28.64)	13 (21.31)	4 (11.43)	33 (18.23)
Miyalgia, *n* (%)	21 (2.76)	107 (18.13)	7 (11.47)	10 (28.57)	42 (23.20)
Headache, *n* (%)	59 (7.75)	92 (15.59)	7 (11.47)	9 (25.71)	39 (21.55)
Malaise, *n* (%)	9 (1.18)	24 (4.07)	1 (1.64)	2 (5.71)	7 (3.87)
Fever, *n* (%)	5 (0.66)	56 (9.49)	3 (4.92)	5 (14.28)	35 (19.34)
Chills, *n* (%)	2 (0.26)	15 (2.54)	3 (4.92)	6 (17.14)	19 (10.50)

*Note:* Frequency and distribution of reported AEFI for participants immunized with various primary and booster vaccination regimens, including CoronaVac, Comirnaty, Covishield, and Ad26.COV2.S.

### 3.3. Breakthrough Infection

During the study, 232 nasopharyngeal swabs were collected; 29, 31% (68/232) tested positive, including one reinfection, while 70, 69% (164/232) were negative. Among positives, 45, 59% (31/68) met the criteria for referral to NGS (Cycle Threshold ≤ 34 in RT‐qPCR). Of these, 58% (18/31) corresponded to the Omicron variant, 35.5% (11/31) to Delta, and 6.5% (2/31) to Gamma. Detailed variant distribution is shown in Supporting figure 3.

Among the 68 COVID‐19‐positive samples, 33 occurred in July 2021, 14 in February 2022, 5 in November 2021, 4 in May 2022, 3 in August 2022, and 9 in November 2022.

### 3.4. Vaccine Immunogenicity

#### 3.4.1. Anti‐SARS‐CoV‐2 S IgG Titers

The IgG anti‐S total antibody response remained above the established cut‐off (OD = 0.1508) throughout the entire follow‐up period, with the majority of participants remaining seropositive at all evaluated time points, indicating the persistence of detectable humoral immune responses over time. An increase was observed following the first booster dose. Although the majority of participants remained seropositive throughout follow‐up, a significant decline in seropositivity was observed at the 9–12 and 15–18 months time points, evidenced by both a reduction in the proportion of individuals with anti‐Spike IgG levels above the established cut‐off and a statistically significant decrease compared with the preceding follow‐up period (both *p* < 0.0001) Figure 1.

**FIGURE 1:**
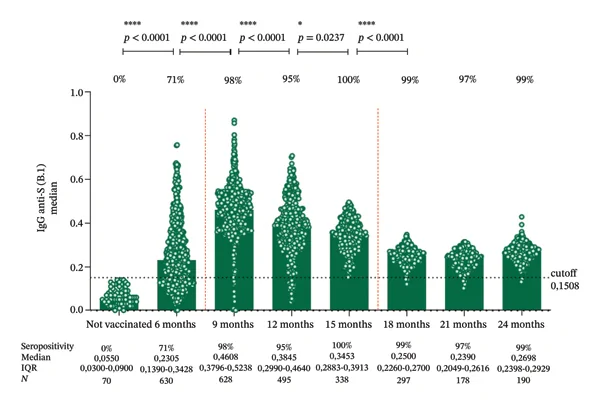
Determination of SARS‐CoV‐2 anti‐S IgG ELISA seropositivity. The median anti‐S IgG OD is represented by the bars. Individual dots show the OD of each sample collected at specific time points after vaccination. The cutoff point of 0.1508 is represented by the black dashed line. Statistical differences with significance levels between groups (*p* < 0.05) are indicated by asterisks. The red dashed lines mark the introduction of the first and second booster doses in the vaccination protocol, respectively. Seropositivity values, median, interquartile ranges (IQR), and sample size (*N*) are detailed in the table below the graph.

Figure 1 includes only participants who received Comirnaty as the first booster. The 19 participants who received Covishield and the 10 who received Ad26COV2.S as the first booster were excluded.

Between individuals with one COVID‐19 infection (either before or after vaccination), a difference was observed between those immunized with PP and PP + Comirnaty at 9 months after the PP, which is the same time point when the first booster was introduced in the vaccination protocol. No difference was found between those immunized with one booster and those with two boosters Figure 2A. In comparison between individuals without a previous COVID‐19 infection, the same pattern was observed Figure 2B.

**FIGURE 2:**
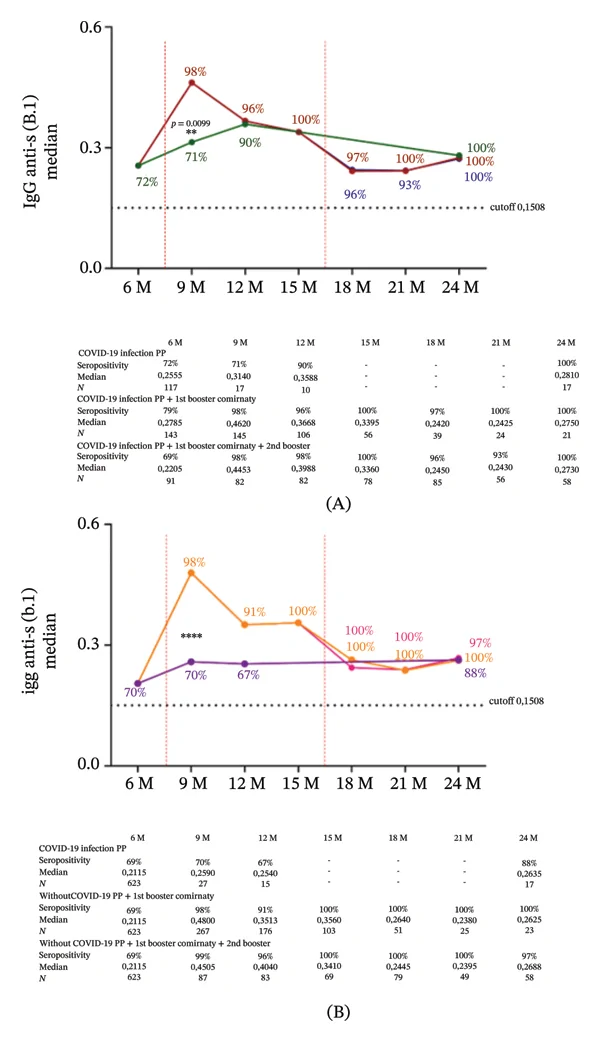
Comparison of anti‐S IgG seropositivity after vaccination between individuals with or without COVID‐19 infection with different vaccination protocols. (A) Individuals with a history of COVID‐19. The green line represents the median OD of the group immunized with CoronaVac PP. The red line represents the median OD of the group immunized with CoronaVac PP + Comirnaty as the first booster, and the blue line represents the median OD of the group immunized with CoronaVac PP + Comirnaty as the first + second booster. (B) Individuals without COVID‐19. The purple line represents the median OD of the group immunized with CoronaVac PP. The orange line represents the median OD of the group immunized with CoronaVac PP + Comirnaty as the first booster, and the pink line represents the median OD of the group immunized with CoronaVac PP + Comirnaty as the first + second boosters. Individual dots indicate the percentage of seropositivity for each time point after vaccination. The cutoff point of 0.1508 is represented by the black dashed lines. Statistical significance (*p* < 0.05) is indicated by asterisks, which denote comparisons between groups at the same time point. Two asterisks indicate *p* = 0.0099, whereas four asterisks indicate *p* < 0.0001. The red dashed lines mark the introduction of the first and second booster doses in the vaccination protocol, respectively. Seropositivity values, median, and *N* of samples are detailed in the tables below the graphs.

When comparing seropositivity between individuals with no history of COVID‐19 and those infected prior to vaccination, a significant difference was observed at 6 months post‐PP, which disappeared following the administration of the first booster (Supporting figure 4A). No differences were observed between age groups of 18–45 and 46–77 (Supporting figure 4B), nor between genders (Supporting figure 4C).

#### 3.4.2. Viral Neutralization Test

In comparison between groups with and without COVID‐19 infection, those with previous infection had higher levels of antibodies neutralizing the Omicron (BA.1) variant, especially at 6 and 12 months after the CoronaVac PP. The first booster significantly increased these levels in both groups. The second booster enhanced the response in uninfected individuals and maintained it in previously infected ones. After the second booster, all participants showed significant neutralizing activity sustained up to 24 months Figure 3.

**FIGURE 3:**
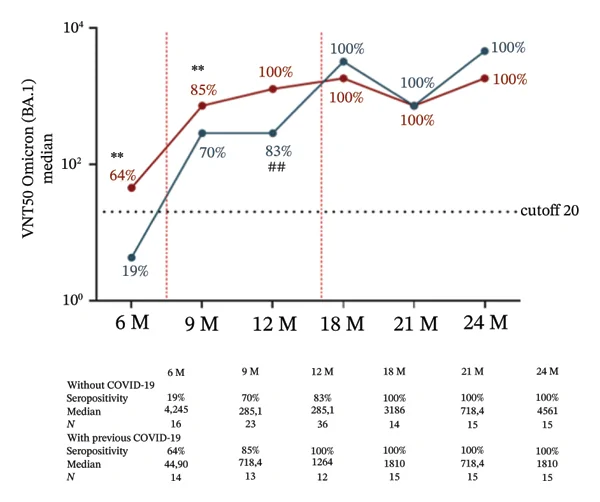
Comparison of viral neutralization of the Omicron (BA.1) variant in participants without prior COVID‐19 infection (represented by the blue line) and participants with prior COVID‐19 infection (represented by the red line). Individual points indicate the percentage of viral neutralization capacity at each time point after vaccination. The cutoff point of 20 is represented by the black dashed line. Statistical significance (*p* < 0.05) is indicated by asterisks and hash marks. Asterisks denote comparisons between groups at the same time point (*p* = 0.0053 and *p* = 0.0041, respectively), whereas hash marks denote comparisons between different time points within the same group (*p* = 0.0084). The red dashed lines represent the introduction of the first and second booster doses, respectively. Seropositivity values, median, and *N* of samples are detailed in the table below the graph. Statistical differences with significance levels between groups (*p* < 0.05) are indicated by asterisks.

#### 3.4.3. Determination of Immunological Biomarker Profile

Radar graph analysis showed greater expansion of soluble mediators between 6 and 15 months after the CoronaVac PP in individuals with prior COVID‐19 infection compared to those who did not have COVID‐19. After 18 months and following the second booster, this index increased, became equal between both groups, and remained high until 24 months. In the group without prior infection, biomarker levels declined following the first booster and continued decreasing until 12 months Figure 4. Correlation analysis revealed that from 6 to 15 months, the group without prior infection exhibited more biomarker interactions (more robust response). However, after the introduction of the second booster dose at 18 months, the group with prior COVID‐19 infection showed more interactions. At 21 and 24 months, the uninfected group again exhibited more interactions Figure 5. Regarding the kinetics of each cellular response biomarker, all markers peaked between 15 and 18 months post‐PP Figure 6A. Until 15 months, biomarker levels were higher in previously infected individuals. Levels of CCL4, CCL2, CCL10, TNF‐α, IFN‐γ, IL‐15, IL‐17, IL‐4, IL‐5, and IL‐2 were consistently higher in the prior infection group at all evaluated time points compared to the group without COVID‐19 infection Figure 6B.

**FIGURE 4:**
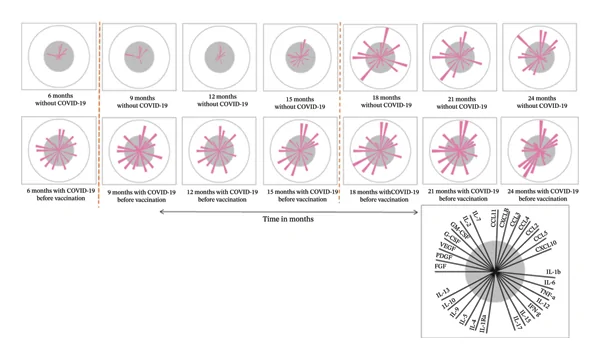
Radar chart illustrating the timeline kinetics of serum biomarkers in participants vaccinated with CoronaVac PP plus heterologous booster doses in groups with and without prior COVID‐19 infection. The pink rays represent the cross‐sectional analysis of chemokine levels (CXCL8, CCL11, CCL3, CCL4, CCL2, CCL5, and CXCL10); pro‐inflammatory cytokines (IL‐1b, IL‐6, TNF‐a, IL‐12, IFN‐g, IL‐15, and IL‐17); regulatory cytokines (IL‐1Ra, IL‐4, IL‐5, IL‐9, IL‐10, and IL‐13); and growth factors (FGF‐basic, PDGF, VEGF, G‐CSF, GM‐CSF, IL‐7, and IL‐2) in serum samples. The gray circle represents the 50% value, and the larger circle marks 100%. The red dashed lines indicate the time points of the first and second booster doses, respectively. The radar chart displayed in the lower right corner serves as a reference template for interpreting individual radar plots. Each radial axis corresponds to one of the evaluated soluble mediators, identified by its respective acronym, facilitating the visualization and interpretation of biomarker distribution across all radar charts.

**FIGURE 5:**
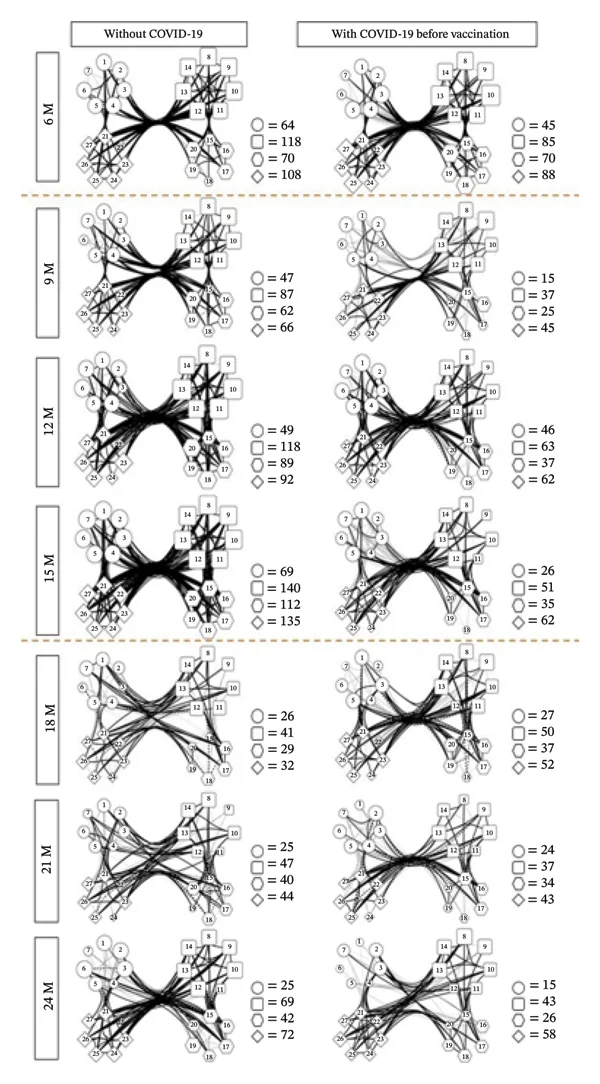
Correlation analysis presented as a network of chemokines (circle), pro‐inflammatory cytokines (equilateral triangle), regulatory cytokines (diamond), and growth factors (hexagon) over time after vaccination. Each parameter is represented by a node. The red dashed lines indicate the introduction of the first and second booster doses, respectively. Statistical correlations (*p* < 0.05) are shown by a line connecting the two nodes. The correlation was classified as weak (*r* < 0.36), moderate (0.36 < *r* < 0.68), and strong (*r* > 0.68), based on the absolute value of the correlation index (*r*), represented by the line thickness. Thick edges and thin connecting edges represent strong and moderate correlations between soluble mediators, respectively. Positive correlation is shown by a solid line, while negative correlation is represented by dashed lines. The number of connections is also expressed.

**FIGURE 6:**
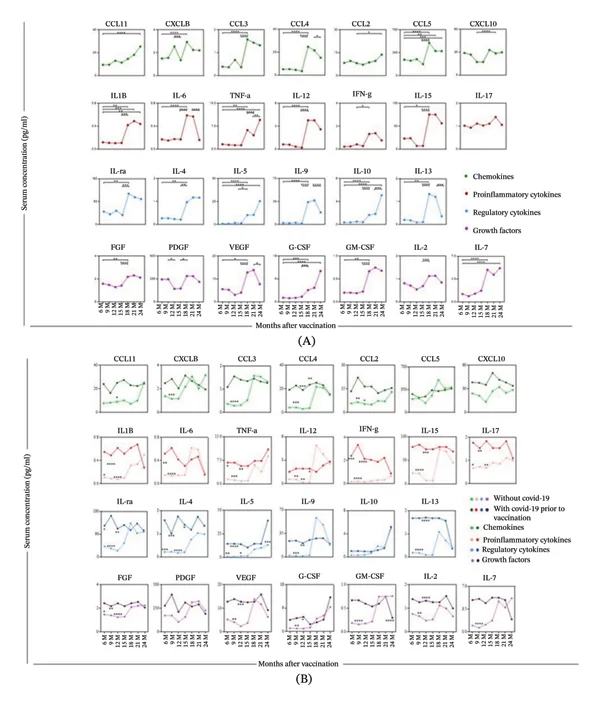
Profile of serum soluble mediators following SARS‐CoV‐2 vaccination with two doses of CoronaVac and heterologous booster doses. The figures display the profile of serum soluble mediators over the study period (6 to 24 months post‐primary protocol [PP]) in individuals vaccinated with two doses of CoronaVac followed by heterologous booster doses. Statistical differences are indicated by asterisks (significance level *p* < 0.05). (A) Profile of serum soluble mediators over the period from 6 to 24 months after the primary protocol. (B) Comparison of the profile of serum soluble mediators between individuals who remained infection‐naive during the study and those with a prior SARS‐CoV‐2 infection preceding the CoronaVac PP.

## 4. Discussion

Initiated concurrently with the COVID‐19 vaccination campaign in Brazil in February 2021, this study demonstrated the effectiveness of vaccination in inducing immune responses against SARS‐CoV‐2. It further assessed the impact of distinct immunization regimens, including different dose schedules and vaccine platforms, on immunogenicity and overall efficacy.

The literature indicates that the adverse events following vaccination reported for COVID‐19 have generally been mild to moderate and did not persist beyond a few days [15, 16]. This observation was also noted in this study, where AEFIs were mild and transient, with no serious adverse events (SAEs).

Higher levels of reactogenicity have been associated with viral vector vaccines, as demonstrated in studies by Silva et al., the State Health Surveillance Center of Rio Grande do Sul, and Rolfes et al., which reported increased AEFI linked to Covishield and other viral vector platforms [17–19]. These findings are consistent with the present study, which also observed greater reactogenicity with Ad26.COV2.S and Covishield compared to other vaccines.

Two peaks of RT‐qPCR‐confirmed COVID‐19 cases were observed in this study: the first around July 2021, coinciding with a national surge in cases and deaths [20], and the second around February 2022, during the predominance of the Omicron variant, which reversed the declining trend of the pandemic [21].

In the present study, significant levels of anti‐S IgG antibodies were observed at all time points. However, after the initiation of the first heterologous booster dose for all participants, antibody levels increased significantly. The literature supports that booster vaccinations enhance protection against COVID‐19 [22]. In a study conducted by Clemens and collaborators, comparing a first heterologous booster with a homologous booster in individuals vaccinated with the CoronaVac PP, all heterologous regimens elicited superior anti‐S IgG responses compared to homologous boosters [10].

This analysis also revealed that after the second booster, IgG antiS antibody levels decreased significantly. However, 99% of participants maintained antibody levels above the Cutoff 9 months after the second booster. These findings align with the existing literature, which reports a 1.5‐fold reduction in antibody levels approximately 10 months after the second booster, yet the levels remain protective [23].

At 9 months, coinciding with the introduction of the first booster, anti‐S ELISA levels differed between CoronaVac PP recipients and those who received a Comirnaty booster, both in infection‐naive participants and in those with prior COVID‐19 infection, reinforcing the importance of heterologous boosting. Although no significant difference was observed between one and two booster doses, the immune response remained high up to 24 months, suggesting the first booster induces peak immunity while the second helps maintain it, as supported by the literature [24, 25].

Within 6 months of immunization, previously infected individuals showed higher anti‐S IgG levels compared to infection‐naive participants. These findings are consistent with data from other studies [26–29]. In one such study, Papaneophytou and colleagues found that in individuals vaccinated without a prior SARS‐CoV‐2 infection, anti‐S IgG antibodies were detected in 91.2% of participants, while in the group of vaccinated individuals with prior infection, anti‐S IgG antibodies were detected in 100% of the participants [26].

It was observed that prior SARS‐CoV‐2 infection enhanced the humoral immune response; however, its duration and protective capacity remain unclear [26]. A study has shown that vaccination elicited a stronger response than mild infection [30], underscoring its importance regardless of prior exposure.

After the first booster dose, seropositivity no longer differed between groups with or without prior infection. Srivastava and colleagues similarly demonstrated that booster vaccination equalized differences in antibody responses between previously infected and infection‐naive individuals, supporting our findings and suggesting that prior infection does not confer a greater long‐term immunological response than vaccination [31]. This is significant because vaccination remains a key strategy for protecting against severe disease and reducing the transmission and infection rates, while SARS‐CoV‐2 infection can manifest a wide range of clinical outcomes, from asymptomatic to symptomatic, including severe cases [32–34].

Some studies have shown no significant sex‐based differences in total antibody responses to vaccines, consistent with the findings of the present study [35, 36]. In contrast, previous literature suggests that age may impact immunogenicity, with older individuals typically exhibiting weaker responses [36–38]; however, this trend was not observed here. This discrepancy may be attributed to differences in cohort composition, as prior studies included a substantial proportion of elderly participants (up to 99 years old), whereas the current study had only 2% (*n* = 35) of participants aged ≥ 60 years, despite covering a wide age range (18–77 years). The stronger immune responses observed in younger individuals, who are generally known to respond more robustly to vaccination, were more evident in this cohort.

In the neutralization assays, the Omicron BA.1 variant was employed due to its high incidence and predominance in Brazil during the study period, as further confirmed by sequencing data from this study [39]. Previously infected individuals showed a stronger initial response, consistent with the findings of Sette and Crotty, who reported that neutralizing antibody titers and the breadth of SARS‐CoV‐2 variant recognition were higher in previously infected individuals who received at least one dose of an RNA vaccine against SARS‐CoV‐2. Their study showed that hybrid immunity offers stronger protection against COVID‐19 than infection‐ or vaccine‐induced immunity alone [40].

Following the first heterologous booster dose, a marked increase in neutralizing antibodies was observed in both groups analyzed in this study, consistent with findings by Yue et al., who demonstrated superior efficacy of a three‐dose regimen over two doses [41]. Additionally, another study reinforced the importance of a heterologous booster in enhancing the neutralizing response against SARS‐CoV‐2, further corroborating the findings of the present study [42].

In the present study, the second booster was essential for enhancing the immune response in individuals without prior COVID‐19 infection, in agreement with Regev‐Yochay et al., who reported a significant increase in neutralizing antibody titers against the Omicron B.1.1.529 variant and other strains following a fourth vaccine dose [25, 43].

In this study, no difference in neutralizing antibody response was observed between previously infected and uninfected individuals following the second booster, with both groups maintaining high responses for up to 24 months. The findings suggest that prior infection did not confer superior long‐term neutralization compared to vaccination, consistent with anti‐S ELISA data showing a more favorable immune response induced by vaccination. Another possible explanation, supported by recent literature, is a plateau effect following repeated exposure to the SARS‐CoV‐2 Spike antigen through vaccination and/or natural infection, whereby the humoral immune response reaches a maximal level, and subsequent antigenic exposures primarily contribute to maintaining, rather than substantially increasing, circulating neutralizing antibody levels [44].

In the present study, the results regarding cellular response markers indicated that prior infection with SARS‐CoV‐2 led to a greater release of cellular response biomarkers compared to individuals without prior infection, between 6 and 15 months after the CoronaVac PP. These findings are supported by Naranbhai and collaborators, who observed that individuals with prior infection exhibited higher T‐cell responses, suggesting that repeated antigen exposure may enhance T‐cell responses [45]. Additionally, Payne and collaborators identified that prior SARS‐CoV‐2 infection amplified and accelerated the T‐cell response [46].

Among participants without prior COVID‐19 infection, a decline in biomarker levels was observed between 6 and 12 months, including after the first heterologous booster dose. Nonetheless, approximately four months after the first booster (12 months post‐vaccination), correlations between biomarkers increased, indicating a more robust cellular response sustained up to 24 months. This pattern repeated after the second booster, with reduced interactions at 18 months followed by an increase at 21 months—again, about 4 months after the booster—with sustained cellular responses through 24 months.

One hypothesis is that vaccine‐induced immunogenicity peaks over time, and the full immune response may not be evident in the short term. This is supported by Voysey et al., who reported that individuals aged 18 to 55 years showed over a twofold increase in binding antibody responses when the interval between COVID‐19 vaccine doses was ≥ 12 weeks, compared to intervals < 6 weeks [47]. These findings highlight the importance of booster doses in enhancing cellular responses to SARS‐CoV‐2.

Grenfell et al. assessed the immunogenicity of the CoronaVac PP followed by a heterologous Comirnaty booster and observed a strong release of cellular immune mediators within the first month, which gradually declined, with no significant increase postbooster. Nonetheless, enhanced interaction among mediators over time suggested a more robust cellular response following the heterologous booster [1]. These findings are consistent with the present study and underscore the importance of heterologous boosters in strengthening the immune response to COVID‐19.

In this study, the difference in biomarker release between individuals without prior COVID‐19 infection and those with infection prior to vaccination disappeared following the introduction of the second booster, and a significant increase in cellular response markers was observed in both groups. This finding aligns with a study by Munro and collaborators, which demonstrated that T‐cell responses were enhanced after the second booster, surpassing the maximum responses observed after the third dose, thereby reaffirming the data presented in this study [48].

Notably, this increase in the cellular response induced by the second booster was not reflected in the total antibody response. As noted by Papaneophytou and collaborators, the humoral response—antibodies—is not the sole mechanism of protection against SARS‐CoV‐2 and other infections. Cellular‐mediated immunity, such as T‐cell responses, can persist even in the absence of detectable total antibodies in serum samples [26].

An important consideration in interpreting these data is the timing of sample collection relative to external factors. The 15‐ and 18‐month collections occurred in May and August 2022, respectively, overlapping with Belo Horizonte’s influenza vaccination campaign, which began in April 2022 and targeted healthcare workers and the elderly [49]. This temporal coincidence suggests that the increase in cellular response biomarkers observed between these time points may reflect not only responses to SARS‐CoV‐2 vaccination but also potential immunological stimulation from the influenza vaccine or other concurrent exposures.

The correlation analysis of cellular response biomarkers revealed that participants without prior SARS‐CoV‐2 infection exhibited a more robust cellular response at nearly all time points, except at 18 months. These findings suggest that prior infection did not enhance biomarker correlation or cellular immune responses. As no similar analyses have been reported in the literature, this study contributes novel insights into the immunogenicity induced by SARS‐CoV‐2 vaccination.

A limitation of this study is that the circulating cytokine and chemokine concentrations measured at the later follow‐up time points may have been influenced by multiple biological and environmental factors, including concurrent infections, lifestyle‐related variables, additional vaccinations, as mentioned in the paragraph above regarding the influenza vaccination campaign, and normal interindividual variability. Although these potential confounding factors cannot be completely excluded, the consistent temporal patterns observed across the study population are unlikely to be explained solely by random individual variation. Rather, these findings likely reflect the long‐term immunological landscape resulting from the combined effects of vaccination, prior SARS‐CoV‐2 infection, and the dynamics of immune memory, consistent with previous reports discussed herein. Nevertheless, a more direct assessment of long‐term vaccine‐induced cellular immunity would require complementary functional approaches, such as antigen‐specific cell stimulation followed by intracellular cytokine analysis or detailed immunophenotyping, which were beyond the scope of the present study.

Another limitation of this study is the challenge of tracking all infections and vaccinations over time, as some cases may be asymptomatic or unreported by participants. These unreported infections and vaccinations could influence the immune response and affect certain analyses. An additional limitation is participant attrition over the study period, despite the inclusion of new participants at each analysis time point. This weakness was further exacerbated by the fact that the study was conducted in a hospital setting, which experienced considerable staff turnover throughout the two‐year period. The frequent rotation of healthcare professionals made it difficult to ensure continuity in participant follow‐up, hindering the implementation of a stable longitudinal protocol and contributing to participant loss.

However, the present study demonstrates the importance of analyzing a wide variety of individuals for a long period in a country with great genetic variability.

## 5. Conclusions

The results indicate that COVID‐19 vaccines are safe and effective in inducing a robust and lasting humoral and cellular immune response, with significant levels of total IgG anti‐S antibodies and neutralizing antibodies against the BA.1 variant observed over a 2‐year period. The administration of heterologous booster doses is crucial for the enhancement and maintenance of these responses. These findings underscore the importance of full vaccination protocols, including the introduction of booster doses, for effective and long‐lasting protection against COVID‐19.

## Author Contributions

Sarah Vieira Contin: methodology, patient follow‐up, data collection, writing, literature review, editing. Nathalie Bonatti Franco Almeida: methodology, patient follow‐up, data collection. Priscilla Soares Filgueiras: methodology, patient follow‐up, data collection. Camila Amormino Corsini: methodology, patient follow‐up, data collection, review. Daniel de Miranda: methodology, patient follow‐up, data collection. Wander de Jesus Jeremias: methodology. Olindo Assis Martins‐Filho: data analysis, conceived and designed the experiments. Andrea Teixeira‐Carvalho: data analysis. Ana Carolina Campi‐Azevedo: methodology and data analyses. Dayane Andriotti Otta: methodology and data analyses. Jaquelline Germano de Oliveira: data analyses. Guilherme Rodrigues Fernandes Campos: methodology and data analysis. Mauricio Lacerda Nogueira: conceived and designed the experiments. Pedro Augusto Alves: data analyses, conceived and designed the experiments. Thaís Bárbara Silva: methodology and data analyses. Gabriel da Rocha Fernandes: methodology and data analyses. Leda Castilho: conceived and designed the experiments. Immunita‐001 Team: methodology, patient follow‐up, data collection. Rafaella Fortini Queiroz Grenfell: conceptualization, supervision, project administration, funding acquisition, resources, writing–review and editing.

## Funding

This research was funded by Oswaldo Cruz Foundation (FIOCRUZ) to RG and scholarship to CAC, DAO, ELO, AESL, AJL, and PFSM. Coordination for the Improvement of Higher Education Personnel–Brazil (CAPES)–Finance Code 001 scholarship to SVMC, NBFA, DAPM, and JVA. Minas Gerais State Research Support Foundation (FAPEMIG) scholarship to PSF. São Paulo State Research Support Foundation (FAPESP) scholarship to GRFC.

## Disclosure

All authors have read and agreed to the published version of the manuscript. A preprint has previously been published [50].

## Ethics Statement

This study was analyzed and approved by the National Research Ethics Commission (CONEP: CAAE 30428720.3.0000.5091).

## Consent

Informed consent was obtained from all subjects.

## Conflicts of Interest

The authors declare no conflicts of interest.

## Supporting Information

Additional supporting information can be found online in the Supporting Information section.

## Supporting information


**Supporting Information** The following supporting information can be found online: Supporting Figure 1: Flowchart of the study methodology, outlining participant recruitment, sample collection, laboratory analyses, and follow‐up procedures. Supporting Figure 2: Vaccination flowchart detailing the distribution of primary and booster doses among participants at the two study hospitals. Supporting Figure 3: Diagram of SARS‐CoV‐2 infection outcomes and variant distribution based on RT‐qPCR and NGS sequencing results. Supporting Figure 4: Comparative kinetics of anti‐S IgG seropositivity according to prior infection status, age group, and sex, including median optical density values and statistical comparisons over time.

## Data Availability

The data that support the findings of this study are available from the corresponding author upon reasonable request.
