# Supplementary material for: Long‐Term Immunogenicity, Safety, and Reactogenicity of CoronaVac and Heterologous Booster Vaccines: A Two‐Year Cohort Study in Brazil
Source: Can J Infect Dis Med Microbiol. 2026 Nov 9;2026:3683298. doi: 10.1155/cjid/3683298 (PMC13746319; doi:10.1155/cjid/3683298)
Supplement: Supplementary file 1 [file CJID-2026-3683298-s001.docx]

**Supplementary figure 1.** Detailed flowchart illustrating the study's methodology and procedural steps. Participants were recruited at partner hospitals (1), followed by the signing of the ICF and completion of questionnaires (2). Blood samples were collected (3) and analyzed for anti-S IgG levels using ELISA (4), immunological biomarkers via Luminex assay (5), and viral neutralization through VNT50 (6). Participant follow-up was conducted via phone (7). For those reporting symptoms, nasopharyngeal swabs were collected (8), and SARS-CoV-2 RNA was extracted and purified (9). The presence of SARS-CoV-2 was confirmed using RT-qPCR (10), and positive samples underwent genome sequencing through NGS (11). Study data were compiled and submitted to health managers (12).

**Supplementary figure 2.** Vaccination flowchart of participants at Hospital da Baleia and Hospital Metropolitano Doutor Célio de Castro, Belo Horizonte, Minas Gerais, including primary and booster doses administration.

**Supplementary figure 3.** Flowchart of SARS-CoV-2 infections and variant distribution. Number of nasopharyngeal swab samples processed by RT-qPCR, results of RT-qPCR testing, and NGS sequencing of positive samples, including variant identification.

**Supplementary figure 4.** Comparison of anti-S IgG seropositivity kinetics following covid-19 vaccination based on prior infection, age group, and sex. The figure displays the percentage of anti-S IgG seropositivity (individual dots) and the median optical density (OD) values for each group over time after vaccination. The cut off point for seropositivity (OD = 0.1508) is indicated by a black dashed line. The introduction of the first and second booster doses is marked by red dashed lines. Statistical significance (p < 0.0001) is indicated by four asterisks. Seropositivity values, median OD, and sample sizes (N) at each time point are provided in the table associated with the graph. (A) Comparison of seropositivity between participants without prior covid-19 infection (pink line, median OD) and those with prior infection before vaccination (green line, median OD). Statistical significance (p<0.05) between the groups is indicated by asterisks. (B) Comparison of seropositivity between the 18–45-year-old group (red line, median OD) and the 46–77-year-old group (blue line, median OD). (C) Comparison of seropositivity between female (purple line, median OD) and male participants (green line, median OD).
